# Analysis of the Foaming Window for Thermoplastic Polyurethane with Different Hard Segment Contents

**DOI:** 10.3390/polym13183143

**Published:** 2021-09-17

**Authors:** Mercedes Santiago-Calvo, Haneen Naji, Victoria Bernardo, Judith Martín-de León, Alberto Saiani, Fernando Villafañe, Miguel Ángel Rodríguez-Pérez

**Affiliations:** 1 Cellular Materials Laboratory (CellMat), Condensed Matter Physics Department, Faculty of Science, University of Valladolid, 47011 Valladolid, Spain; jmadeleon@fmc.uva.es (J.M.-d.L.); marrod@fmc.uva.es (M.Á.R.-P.); 2 Department of Chemical Engineering, Faculty of Engineering, University of Babylon, Hilla 51001, Iraq; eng.haneen.zuhair@uobabylon.edu.iq; 3 CellMat Technologies S.L. Paseo de Belén, 47011 Valladolid, Spain; vbernardo@fmc.uva.es; 4 School of Materials, The University of Manchester, Oxford Road, Manchester M13 9PL, UK; a.saiani@manchester.ac.uk; 5 GIR MIOMeT-IU Cinquima-Química Inorgánica, Faculty of Science, University of Valladolid, 47011 Valladolid, Spain; fernando.villafane@uva.es

**Keywords:** thermoplastic polyurethane, foams, gas dissolution foaming, foaming window, hard segment

## Abstract

A series of thermoplastic polyurethanes (TPUs) with different amounts of hard segments (HS) (40, 50 and 60 wt.%) are synthesized by a pre-polymer method. These synthesized TPUs are characterized by Shore hardness, gel permeation chromatography (GPC), differential scanning calorimetry (DSC), wide angle X-ray diffraction (WAXD), dynamic mechanical thermal analysis (DMTA), and rheology. Then, these materials are foamed by a one-step gas dissolution foaming process and the processing window that allows producing homogeneous foams is analyzed. The effect of foaming temperature from 140 to 180 °C on the cellular structure and on density is evaluated, fixing a saturation pressure of 20 MPa and a saturation time of 1 h. Among the TPUs studied, only that with 50 wt.% HS allows obtaining a stable foam, whose better features are reached after foaming at 170 °C. Finally, the foaming of TPU with 50 wt.% HS is optimized by varying the saturation pressure from 10 to 25 MPa at 170 °C. The optimum saturation and foaming conditions are 25 MPa and 170 °C for 1 h, which gives foams with the lowest relative density of 0.74, the smallest average cell size of 4 μm, and the higher cell nucleation density of 8.0 × 109 nuclei/cm^3^. As a final conclusion of this investigation, the TPU with 50 wt.% HS is the only one that can be foamed under the saturation and foaming conditions used in this study. TPU foams containing 50 wt.% HS with a cell size below 15 microns and porosity of 1.4–18.6% can be obtained using foaming temperatures from 140 to 180 °C, saturation pressure of 20 MPa, and saturation time of 1 h. Varying the saturation pressure from 10 to 25 MPa and fixing the foaming temperature of 170 °C and saturation pressure of 1 h results in TPU foams with a cell size of below 37 microns and porosity of 1.7–21.2%.

## 1. Introduction

Foamed polymers are widely used in almost all industrial sectors, due to their interesting value-added properties, such as low density, low thermal conductivity, and adaptable mechanical properties depending on their relative density. Polyurethane (PU) is the polymer par excellence for foaming mainly because of the high applicability of PU foams, which is based on the possibility of tailoring the final properties of the material by adequately changing the types or quantities of the initial components (isocyanate, polyol, surfactants, catalysts, and blowing agents). Moreover, the simplicity of the PU foaming technology makes these materials very competitive from an economic perspective. PU foams represent 50% of the global foam market [1]. Although most of the PU foams are thermoset, the study of foams based on thermoplastic polyurethanes (TPU) is gaining importance in recent years, because they are elastic thermoplastically processable materials, and also because they can be reprocessed or recycled. Thus, TPU foams are nowadays very interesting materials for several industries, such as furniture, automotive, sportswear, and packaging.

TPUs are block copolymers composed of alternating soft segments (SS) and hard segments (HS), which are produced by the polyaddition reaction of a diisocyanate with a medium molecular weight macrodiol and with a low molecular weight diol, which acts as a chain extender. The presence of SS and HS gives rise to a two-phase microstructure, in which the HS from the isocyanate and the chain extender segregate into semicrystalline domains, whereas the SS from the polyol chains form amorphous domains in which the HS are dispersed [2]. Therefore, HS offers rigidity, and SS provides flexibility to the TPU material. Their ratio and distribution, which are key for the final properties, are determined by a large number of chemical and structural factors, including the characteristics of the raw materials and the polymerization procedure and processing conditions used for their synthesis.

The gas dissolution foaming process is one of the most reliable methods developed in order to produce thermoplastics foams, where carbon dioxide (CO_2_) is normally used as a blowing agent. This technology allows manufacturing foamed samples by using a green solvent (CO_2_), which can be removed leaving no residues nor pollutant compounds. Many studies have been published on the use of this method, and different cellular polymers have been obtained, such as poly(methylmethacrylate) (PMMA), polystyrene (PS), poly(lactic acid) (PLA), polycarbonate (PC), polyethylene (PE), or polypropylene (PP) among others [3,4,5]. Nevertheless, the number of reports dealing with TPU foams obtained by the gas dissolution foaming process using CO_2_ as a blowing agent is still rather limited [6,7,8,9,10,11,12,13,14]. These previous papers clearly show that foaming TPU is complicated, not all TPUs can be foamed, and in addition, the processing window for those materials is narrow. As a result, it is important to understand the foamability behavior of the different types of TPU in which the chemical characteristics changes (chemical structure of the components, soft and hard segments contents, etc.). For that, it is necessary to investigate TPUs in which the chemical features are known since until now, the majority of studies are carried out using commercial TPUs.

Therefore, few studies have focused on the effect of chemical characteristics in the fabrication of TPU foams by the gas dissolution foaming process using CO_2_ [12,13,14,15,16]. Ito et al. [12] studied the effect of the chain length of the SS from 1000 to 3000 g/mol of number-average molecular weight (Mn) and the SS contents from 50 to 80 wt.% (which correspond to HS from 50 to 20 wt.%) on the cellular structure of TPU foams obtained by two-step foaming. They concluded that the solubility of CO_2_ in the SS is considerably higher than that in the HS, and thus, the nucleation and growth of the nuclei occurs in the SS area. Yet et al. [13] used three different approaches to investigate the foaming behavior of TPU by a one-step foaming from previously synthesized TPU: increasing the HS content (37 wt.%, 48 wt.%, and 56 wt.%), replacing the SS type (polytetramethylene ether glycol and poly (1,4-butylene adipate) of molecular weight 1000 g/mol), and adding a graphene nucleation agent. All three approaches were able to produce cell sizes of less than 1 μm. The results of the TPUs with different HS and with different SS types indicate that *Tg* is not the only factor that determines whether the sample can be foamed; both hardness and crystallinity significantly affect the foaming capability of the samples. Ge et al. [14] foamed synthesized TPUs with very low HS contents (26 wt.%, 32 wt.%, and 36 wt.%) by two-step foaming. The cell structure evolution of TPU foams with the foaming temperature and the foaming time showed that the low HS content was beneficial for the expansion ratio because it reduced the unfoamed region, whereas the high HS content generated the microcrystalline region acting as the nucleating site to improve the cell density. Recently, Nofar et al. [15] investigated the microcellular foaming behavior of three different commercial TPU grades that possessed similar SS molecular weight of around 1000 g/mol and contained different HS contents of 38.6 wt.%, 48.9 wt.%, and 56.9 wt.% using one-step pressure drop batch based bead foaming. It was observed that the increase in HS content expanded and shifted the processing windows to higher temperatures and also limited TPU foam expansion but at same time minimized the shrinkage. The same group later [16] continued exploring the foaming behavior of three commercial TPU grades with a given HS content (39 wt.%) but with various SS molecular weights (1000, 2000, and 3500 g/mol) using the previous bead foaming method using supercritical CO_2_. The results showed that the increase in SS length could not only enhance the foam expansion behavior but could also enhance the heterogeneous cell nucleation and more importantly minimize the shrinkage of the TPU foams as a result of broadened HS crystallization.

Considering the previous reports about the effect of the chemical characteristics of TPU on the foaming behaviour in which in many of the TPUs are provided by chemical companies, we have synthesized new TPUs to evaluate in more detail the effect of HS content on the foaming window of TPU. Mainly, the study aims to analysis the relation between the main characteristics of synthesized TPUs with their foamability. In order to fulfil this objective, a new series of TPUs with different characteristics have been synthesized. In particular, we herein report the synthesis of TPUs based on 4,4-diphenylmethanediisocyanate (MDI) and, poly(ethyleneglycol)-block-poly(propyleneglycol-block-poly(ethylen-glycol) (PEG-PPG-PEG) with an intermediate average Mn of ≈2000 g/mol and 1,5-pentanediol (1,5-PDO) components containing different HS contents (40 wt.%, 50 wt.%, and 60 wt.%) by using the pre-polymer method. These TPUs are herein characterized by Shore hardness, GPC, DSC, WAXD, DMTA and rheology. The foaming behavior of these materials is also extensively analyzed at different saturation and foaming conditions by a one-step gas dissolution foaming process. The resultant foams are characterized by measuring the density and cellular structure.

## 2. Materials and Methods

### 2.1. Materials

The reactants used to obtain TPU are as follows: MDI with a molecular weight of 250.25 g/mol and a functionality of 2, PEG-PPG-PEG as a macrodiol with an average Mn of ≈2000 g/mol and OH index of 56–59 mg KOH/g, 1,5-PDO as a chain extender with a molecular weight of 104.15 g/mol and a functionality of 2, 1,4-diazabicyclo[2.2.2]octane (DABCO) as catalyst and N,N-dimethylacetamide (DMAc) as solvent. All of them were supplied by Sigma Aldrich (St. Louis, MO, USA) and used as received, except for PEG-PPG-PEG and 1,5-PDO, which were dried in a vacuum oven overnight at 80 °C and then stored in sealed glass jars with molecular sieves, and DABCO, which was deoxygenated by bubbling nitrogen before use. Medical grade CO_2_ (99.9% purity) was used as a blowing agent for the gas dissolution foaming experiments.

### 2.2. TPU Synthesis

Three samples of TPU were synthesized with different weight fractions of HS: 40% HS, 50% HS and 60% HS (percentage indicated is by weight). The synthesis of TPUs was carried out in a reaction flask by a two-step, pre-polymer method under dry nitrogen atmosphere. Firstly, PEG-PPG-PEG was added drop wise from a funnel to an excess of MDI and this mixture was heated in an oil bath to 80 °C for 2 h with stirring at 400 rpm. After this step, a pre-polymer that contains a polyol end capped by MDI is obtained in the presence of an excess of MDI. In the second step, the required amount of the pre-polymer, MDI, and DMAc (240 mL) was stirred vigorously with a magnetic stirrer until the MDI granules were dissolved. Then, the above mixture was poured into a funnel and then added drop wise to a preheated mixture of chain extender (1,5-PDO) and DABCO catalyst (0.3 g, 0.003 mmol, 0.3%) in DMAc (60 mL). This reaction mixture was stirred at 400 rpm for 2 h in an oil bath at 80 °C. At the end of this stage, hard segments were created by the reaction of the MDI with the chain extender. Finally, the solution containing the TPU polymer was poured into silicone moulds and maintained in an oven at 80 °C for 3 days to obtain TPU casts.

The amount of each component used for the different TPUs is collected in Table 1. The molar ratio of NCO/OH was adjusted as 1.02. The pre-polymer formulation (first step of synthesis) used is calculated having a molar ratio of the number of moles of MDI and PEG-PPG-PEG polyol as 6:1 for TPU 50% HS and TPU 60% HS, and as 4:1 for TPU 40% HS. In total, 100 g of TPU material were produced in each synthesis.

### 2.3. Samples Roduction of Solid TPU

Compression molded samples were prepared by using a hot plate press. The material was first heated at a temperature above melting temperature (between 165 and 180 °C) for 3 min, raising the pressure to 100 bar at 0.5 bar/s. Then, the samples were pressed under a constant pressure of 100 bar for 7 min, lowering the temperature to 60 °C at 25 °C/min. These compression molded samples were used to characterize their Shore hardness, WAXD, DMTA, and rheology.

Extruded samples with 1.5 ± 0.5 mm of diameter were also prepared by a TWELVindex extrusion plastometer from ATS Faar (Milan, Italy) using a temperature above melting temperature (between 165 and 180 °C). These extruded samples were prepared to carry out the foaming tests.

All the samples were dried in a vacuum oven overnight at 80 °C before any type of characterization and before the foaming process.

### 2.4. Solid TPU Characterization

#### 2.4.1. Density

The density of the solid TPUs (ρs) (extruded samples) was measured with a gas pycnometer Accupyc II 1340 from Micromeritics (Norcross, GA, USA).

#### 2.4.2. Shore Hardness

Shore A and B hardness tenting was measured with a Bareiss U72 durometer (Bareiss, Baiersbronn, Germany) following the “ISO 868:2003: Plastics and ebonite. Determination of indentation hardness by means of a duromer (Shore hardness)” procedure. The measurements were taken for 1 s at the temperature of 23 °C. Three samples of each material from the compression molded sheet with dimensions 10 × 10 × 2 mm^3^ were used.

#### 2.4.3. Gel Permeation Chromatography (GPC)

Number-averaged molecular weights (*M*_n_), weigh-averaged molecular weights (*M*_w_) and polydispersity index (PDI) were determined by GPC. A dilute solution of 1 mg/mL of synthesized TPU was prepared in tetrahydrofuran (THF) and stirred for 2 h. The solutions were filtered through a 0.2 μm polyamide filter. Three measurements of each synthesized TPU were carried out in order to obtain an average.

#### 2.4.4. Differential Scanning Calorimetry (DSC)

DSC was performed using a DSC30 Mettler Toledo Instrument (Mettler-Toledo, Columbus, OH, USA). Tests were carried out under nitrogen atmosphere at a heating rate of 10 °C min^−1^ from −90 to 220 °C. All DSC measurements were done in aluminum pans with 8 mg of extruded TPU. DSC experiments are carried out by performing a first heating, a cooling, and a second heating to eliminate the thermal history. The HS crystallinity of the TPUs (*X*_c_) has been estimated according to Equation (1), where Δ*H*_m_ is the melting enthalpy measured for each TPU and Δ*H*_om_ is the melting enthalpy for 100% crystalline TPU, which was taken as 155 Jg^−1^ [17].

(1) $$X_{c}\left(\%\right)=\frac{\Delta H_{m}}{\Delta H_{\mathrm{om}}^{\;}}\times 100\%$$

#### 2.4.5. Wide Angle X-ray Diffraction (WAXD)

WAXD patterns were obtained using a PANalytical X’Pert Pro (XRD 5) instrument (Malvert Panalytical, Malvern, UK) employing Cu Kα radiation (λ = 1.54 Å) as the X-ray source, and a voltage of 40 KV and 40 mA of current. The diffraction angle was scanned from 5° to 90°. The samples were cut from the compressed sheet with dimensions 10 × 10 × 2 mm^3^.

#### 2.4.6. Dynamic Mechanical Thermal Analysis (DTMA)

DTMA tests were carried out using a Q800 DMA instrument (TA) in single cantilever mode at a heating rate of 3 °C/min, an amplitude of 15 µm and a frequency of 1 Hz. The samples were cut from the compressed sheet, with dimensions 17 × 6 × 2 mm^3^. Three samples of each sample were measured to obtain an average. The storage modulus (E’), loss modulus (E’’), and tan δ were recorded, and the glass transition temperature (*Tg*) of hard segments (HS) and soft segments (SS) was obtained from the peak tan δ.

#### 2.4.7. Shear Rheology

Cylindrical samples obtained by compression molding with a thickness of 1 mm and a diameter of 25 mm were used for the rheological tests. Rheological measurements were carried out on a strain-controlled rheometer (ARES-G2, TA Instruments, New Castle, DE, USA) with parallel plate geometry (diameter 25 mm, gap 1 mm) under a nitrogen atmosphere. The rheological characterization was performed in frequency sweep tests at 200 °C, a constant strain of 10% and an angular frequency range from 0.1 to 100 rad s^−1^. Strain was chosen in order to measure all the samples in the linear viscoelastic region (LVR), and it was estimated by an initial study through an amplitude sweep test at strains ranging from 0.01% to 1000%, an angular frequency of 1 rad/s and 200 °C.

### 2.5. Gas Dissolution Foaming Experiments

A high pressure vessel (model PARR 4681) provided by Parr Instrument Company with a capacity of 1 L was used for the foaming tests. The maximum temperature and pressure reached by this device are 350 °C and 41 MPa, respectively. The pressure was automatically controlled by an accurate pressure pump controller (model SFT-10) provided by Supercritical Fluid Technologies Inc. (Newark, USA, DE) The vessel is equipped with a clamp heater of 1200 W, and its temperature is controlled by a CAL 3300 temperature controller(CAL control, Hertfordshire, UK). This set up has been used to perform a set of experiments by using a one-step foaming process. Firstly, samples were introduced in the pressure vessel under certain pressure and temperature conditions for the saturation stage. After saturation, pressure was rapidly released and the samples expanded in the pressure vessel after depressurization. Two sets of experiments were performed for the extruded samples. Firstly, the effect of the foaming temperature was analyzed by fixing the saturation pressure to 20 MPa and the saturation time to 1 h, and varying the foaming temperature between 140, 150, 160, 170 and 180 °C. Secondly, the influence of the saturation pressure was evaluated by choosing four different saturation pressures: 10, 15, 20 and 25 MPa at 170 °C for 1 h. Extruded TPU samples were foamed and then were left to desorb the remaining CO_2_ before characterizing their density and cellular structure.

### 2.6. Characterization of TPU Foams

#### 2.6.1. Density

Density of the TPU foams was determined with the water-displacement method based on Archimedes’ principle using a density determination kit with an AT261 Mettler-Toledo balance (Mettler-Toledo, Columbus, USA, OH). The density of TPU foams was measured when all CO_2_ was desorbed. Relative density (ρr) has been calculated by the ratio between the density of TPU foam (ρf) and the density of the solid TPU (extruded samples) with the same chemical composition (ρs).

#### 2.6.2. Cellular Structure

With the aim of maintaining the cellular structure for the microscopic visualization, samples were cooled in liquid nitrogen and fractured. The cellular morphology of the extruded samples was observed by Scanning Electron Microscopy (SEM) with a JEOL JSM-820 microscope (JEOL Ltd., Tokio, Japan). The perpendicular plane of the extrusion direction was examined by SEM after vacuum coating with a gold monolayer. An image analysis technique [18] of SEM micrographs was used to determine the average cell size (Ф) of the cellular structure of the TPU foamed. More than 100 cells of different areas of each cellular material have been used for this analysis.

Cell nucleation density (*N*_0_) [18] has been determined according to Equation (2) where *N_v_* is the cell density, Ф is the average cell size, and ρr is the relative density.

(2) $$N_{0}=\frac{N_{v}}{\rho_{r}}=\frac{6\;\left(1-\rho_{r}\right)}{\rho_{r}\pi {Ф}^{3}}$$

## 3. Results and Discussion

### 3.1. Characterization of Solid TPU

A complete characterization of the TPU samples allows relating the TPU properties with the gas dissolution foamability. The relevant properties of the samples studied are collected in Table 2. The density slightly increases when the HS content increases in the TPU material. The measurement of Shore hardness allows testing the TPU resistance into two categories: Shore A is used for more flexible types of TPU whereas Shore D is referred to more rigid varieties. Table 2 shows that the Shore hardness increases with HS content. TPU 60%HS is not classified in the Shore A scale, thus being the most rigid material of all the synthetized products.

In Table 2, GPC measurements of the samples with different contents of HS show that the molecular weights are higher for TPUs with low contents of HS (40%HS and 50%HS) than for TPU 60%HS. The polydispersity is close to 2 in all the TPUs studied. It is expected that the molecular weight of TPUs increases with HS content. This is because when the HS content is increased, the HS chain length increases, since a large excess of diisocyanate reacts with the chain extender after formation of the pre-polymer [19]. Thus, the above explains the increase in molecular weight when the HS content is increased from 40 to 50 wt.%. However, the TPU with 60 wt.% has the lowest molecular weight. This could be because during the synthesis of TPU, the chain extension step is accompanied by a pronounced increase in viscosity, which probably reduces the mobility of the remaining chain extender molecules and as a consequence can hinder the growth of the molecular weight of the polymer [19].

**Figure 1:**
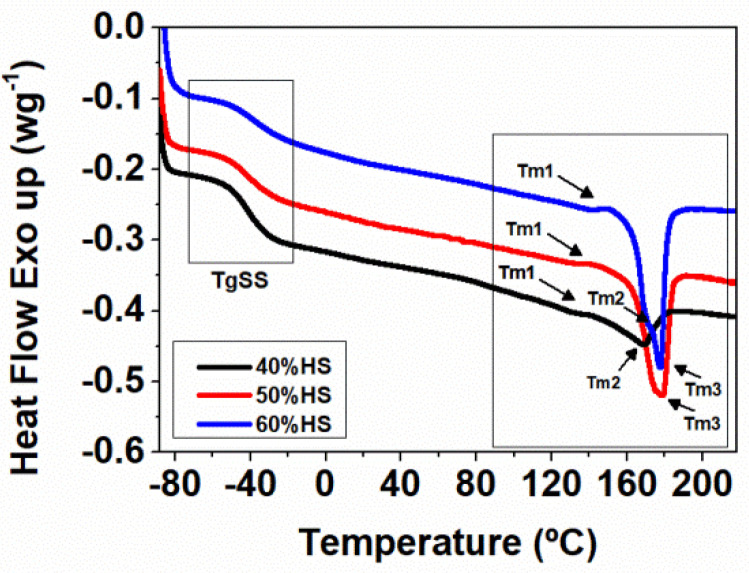
DSC thermograms of the second heating for solid TPUs with different HS contents.

The DSC thermograms of the TPU under study are shown in Figure 1, and the results obtained are collected in Table 3. DSC thermograms of all TPU samples display a first transition at ca. −40 °C associated with the glass transition of the SS (*T*_g_SS). This *T*_g_SS is easily detected due to the high SS contents of the TPUs under study; however, the glass transition of the HS (*T*_g_HS) is not observed. Several endothermic peaks associated with the melting temperatures (*T*_m_ 1–3) of the HS crystalline are observed at higher temperatures, between 130 and 180 °C (Table 3). The observation of several *T*_m_ peaks is related to the different order of crystalline structures. The *T*_m_ of the TPU systems is shifted to higher temperatures when the HS content increases, and the TPU with high HS content (60%HS) presents a sharp endothermic peak at 180 °C, which is related to the formation of highly ordered crystalline structure. Moreover, the HS crystallinity of the samples grows from 7.0% to 9.4% when the HS content increases from 40 wt.% to 60 wt.% (Table 3).

**Figure 2:**
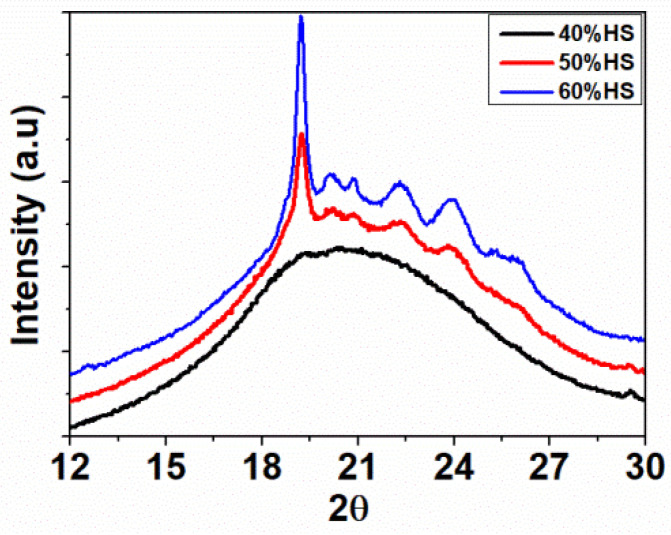
WAXD pattern of solid TPUs with different HS%.

WAXD patterns for TPU systems are shown in Figure 2. The diffraction peaks near 2θ = 19°, 20°, 21°, 22°, 24°, and 26° are obviously attributed to the presence of TPU crystals. The intensity of these peaks increases when TPU samples include more HS contents (50%HS and 60%HS), because these samples present higher crystallinities. In the case of TPU 40%HS, the material is more amorphous, which explains the absent crystalline peaks and, is in agreement with the DSC results.

**Figure 3:**
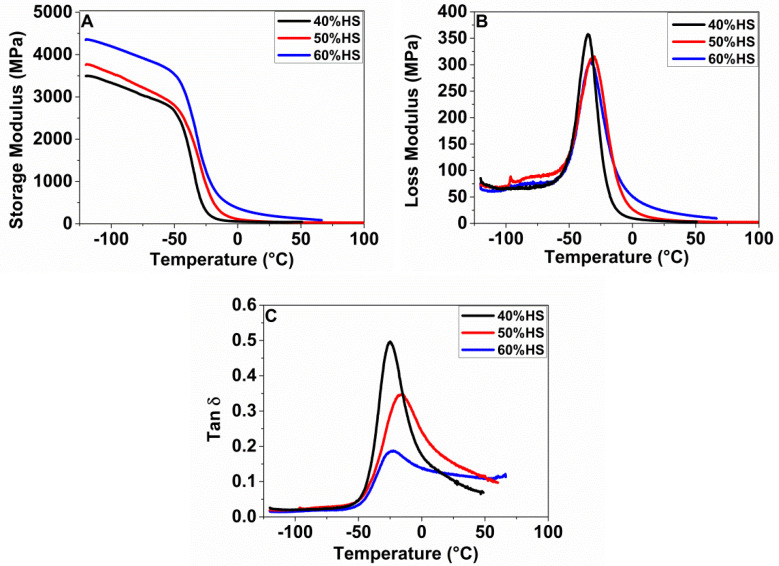
Dynamic mechanical analysis of solid TPUs with different HS%.

**Figure 4:**
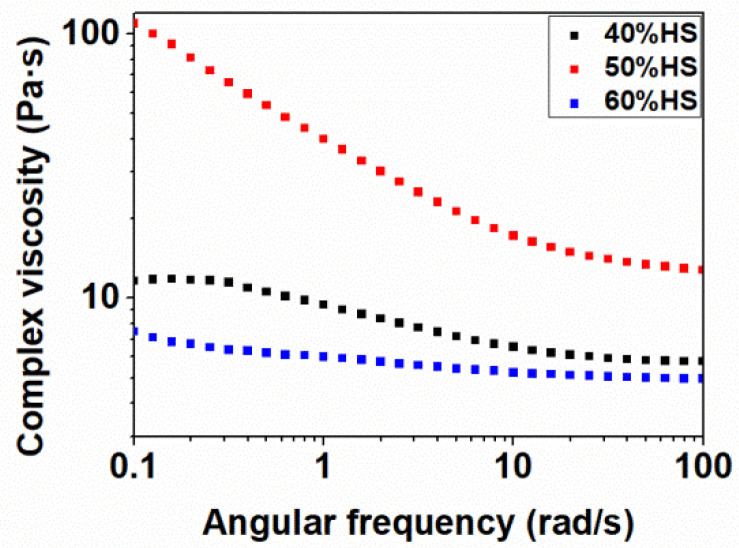
Complex viscosity as a function of the angular frequency for TPUs with different HS%.

The viscoelastic properties of the TPU samples were measured by DMTA. Figure 3 shows the storage modulus, the loss modulus, and damping factor (tan δ), as a function of the temperature for the TPU samples prepared. Table 4 collects the values of *T*_g_SS and the storage modulus at −100 ºC and 25 ºC. As expected, the samples behavior is very different depending on the HS% content. The increase in HS% leads to an increase in the storage modulus and to a decrease in the peak intensity maximum of both the loss modulus and the tan δ [20]. The increase in HS implies an increase in crystallinity, which provides rigidity to the material, and consequently, the storage modulus increases [21]. Moreover, the flattening and broadening of the loss and tan δ peaks are detected with an increase in HS content (samples with 50 and 60 wt.% HS), because of more ordered soft and hard domains, which restrict the amorphous SS molecular mobility [22]. Moreover, the peak of *T*_g_ measured by DMTA shows higher temperatures to those measured by DSC (Figure 4), because of the dependence of the *T*_g_ value with frequency [23], although the trends observed are similar. In addition, as expected, the storage modulus increases below the *T*_g_ (−100 °C) and above the *T*_g_ (25 °C) when the HS content.

A rheological characterization at 200 °C (close to the foaming temperatures used in the paper) was performed for the TPU samples with different HS% to determine the complex viscosity curves shown in Figure 4. TPU 40%HS and TPU 60%HS show small complex viscosities at low frequencies, whereas TPU 50%HS displays a considerably increase in complex viscosity. The difference in melt viscosity becomes smaller at relatively high shear frequency. Clearly, the TPU with 50 wt.% HS greatly increases the melt viscosity in all the angular frequencies with respect to the rest of TPU systems, which could favor its foamability capability. These complex viscosities are in concordance with the molecular weights of each material (Table 2), as the TPU with 50 wt.% HS has higher molecular weight and more complex viscosity than the rest of the materials.

### 3.2. Study of TPU Foaming

The TPUs previously prepared were foamed by a gas dissolution foaming process in one-step, in order to study the effect of HS content (40 wt.%, 50 wt.% and 60 wt.%) on the foaming behavior. We selected low contents in HS since previous studies showed that most of the CO_2_ used for the foaming process is dissolved in SS [12].

**Figure 5:**
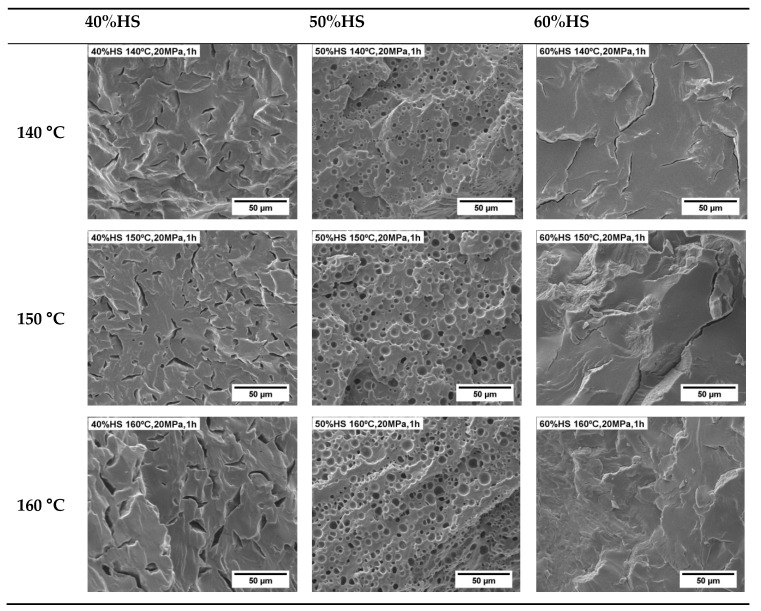

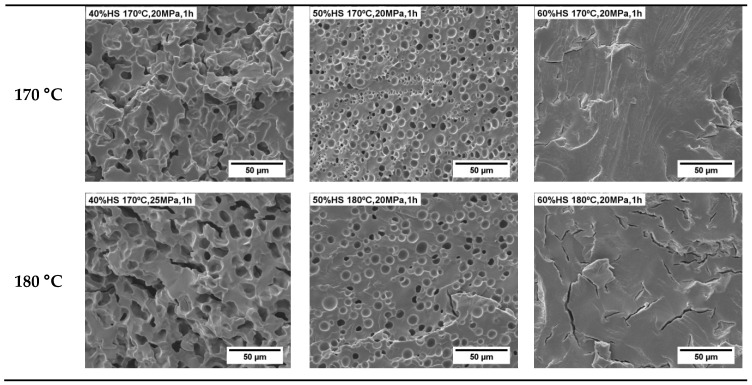
SEM micrographs of TPU with different HS% foamed by a one-step process between 140 and 180 °C, 20 MPa, and 1 h.

In the first part of the study, we evaluated the influence of the foaming temperature by using five different foaming temperatures: 140 °C, 150 °C, 160 °C, 170 °C, and 180 °C. The saturation pressure and foaming time were kept constant at 20 MPa and 1 h, respectively. Figure 5 shows the SEM micrographs of the TPU samples after the foaming process. Only the TPU with 50 wt.% HS shows a stable cellular structure for the different foaming conditions used. The TPU with 60 wt.% HS does not foam, which is probably due to its high HS content, which results in a high crystallinity (DSC results in Table 3 and WAXD pattern in Figure 2) and a high rigidity (Shore harness in Table 2), and also its low viscosity (Figure 4). Conversely, the TPU with 40 wt.% HS with low crystallinity (DSC results in Table 3) gives rise to a highly deteriorated cellular structure, which is clearly seen in Figure 5, especially for high foaming temperatures. The low quality of this cellular structure can be attributed mainly to the low viscosity of the TPU matrix (Figure 4), which could reduce the stability of the cellular structure, thus producing cell coalescence and/or collapsing. Therefore, it is possible that the optimal foaming parameters for the TPUs with 40 wt.% and 60 wt.% have not been found in this investigation and future work is necessary to foam these systems.

**Figure 6:**
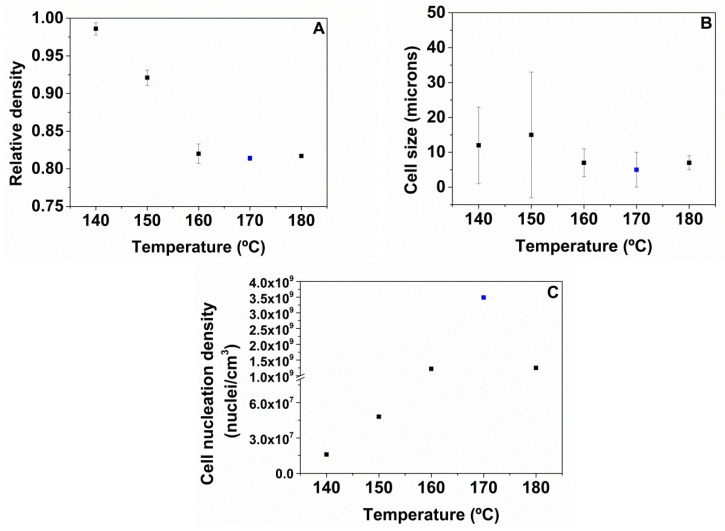
Relative density (**A**), cell size (**B**), and cell nucleation density (**C**) of TPU 50%HS foamed by one-step between 140 and 180 °C, 20 MPa, and 1 h.

Hence, the characteristics of the TPU with 50 wt.% HS are optimum in order to get a better foamability. The relative density, average cell size, and cell nucleation density of the samples foamed with 50 wt.% HS were measured, and the values obtained are summarized in Figure 6. Figure 6A shows that the relative density decreases as the foaming temperature increases, reaching maximum values of expansion at 170 °C. On the one hand, the average cell size decreases when the foaming temperature increases (Figure 5 and Figure 6B), the cellular structure being less homogeneous at lower foaming temperatures (140 °C and 150 °C) possibly due to the crystalline part of TPU not being melted at these temperatures. The TPU foamed at 170 °C displays a small cell size of 5 microns, and a homogeneous cellular structure. The cell nucleation density increases as the foaming temperature increases (Figure 6C), which points to a higher number of cells in the foams produced. A maximum cell nucleation density of 3.5 × 109 nuclei/cm^3^ is reached at 170 °C for all the foaming experiments, which coincides with the lowest cell size of 5 microns and with the lowest relative density of 0.81. These results indicate that the most efficient foaming is reached at 170 °C. At 180 °C, the cell nucleation density is slightly reduced, and consequently, both cell sizes and relative densities slightly increase. These results are a consequence of the softening of the TPU polymer and to a lower viscosity of the system.

**Figure 7:**
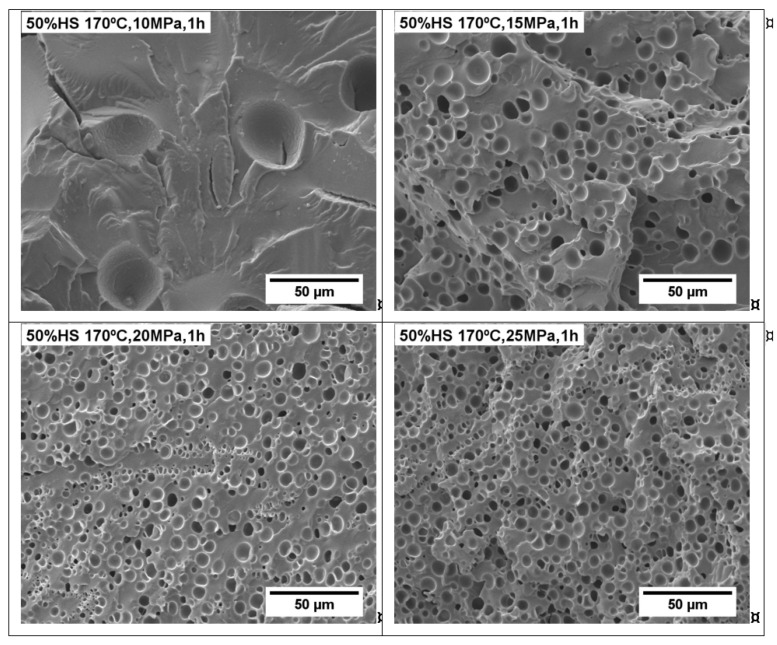
SEM micrographs of TPU 50%HS foamed by one-step between 10 and 25 MPa, 170 °C, and 1 h.

**Figure 8:**
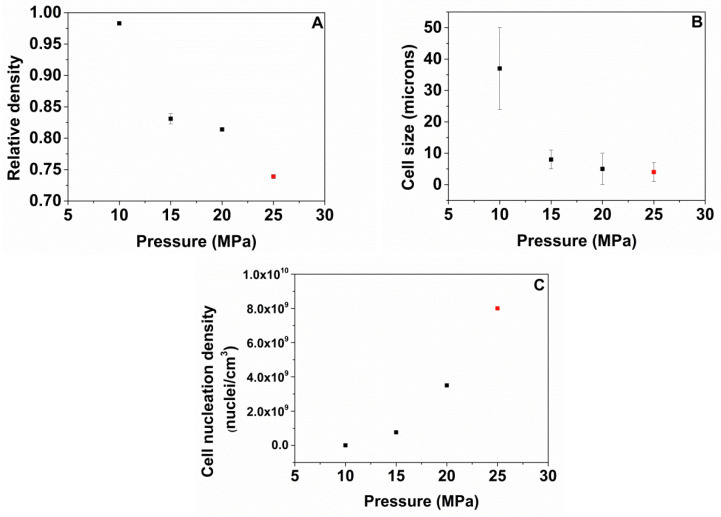
Relative density (**A**), cell size (**B**), and cell nucleation density (**C**) of TPU 50%HS foamed by one step between 10 and 25 MPa, 170 °C, and 1 h.

Since the TPU 50%HS is the optimum content of HS, it was selected in order to optimize the foaming of our TPU system in the second part of this study. For this purpose, the influence of the saturation pressure for TPU 50%HS was evaluated using four different saturation pressures: 10, 15, 20, and 25 MPa, whereas the foaming temperature and foaming time were kept constant at 170 °C and 1 h, respectively. The SEM micrographs and parameters of the TPU foams with 50 wt.% HS are collected in Figure 7 and Figure 8. Figure 8A shows the relative density values, which decrease with the saturation pressure, achieving the lowest relative density when using 25 MPa. Moreover, Figure 8B indicates that a saturation pressure increase leads to important cell size reduction, which is clearly observed in the SEM micrographs shown in Figure 7. Thus, the lowest cell size is obtained for TPU foamed at 25 MPa. All these results are in concordance with the trend of the cell nucleation densities shown in Figure 8C, which increase when increasing the saturation pressure. In general, the increase in the saturation pressure causes a higher amount of gas to be dissolved in the TPU matrix, which leads to smaller relative densities and cell sizes, and also a to a higher cell nucleation density. Nevertheless, the TPU material is scarcely foamed at low saturation pressure (10 MPa), as observed in Figure 7.

In conclusion, the optimum foaming and saturation conditions for the TPU with 50 wt.% HS are 170 °C of saturation pressure, 25 MPa of foaming pressure and 1 h of saturation time. The resulting TPU foam has the lowest relative density (0.74), the smallest cell size (4 microns), and the maximum cell nucleation density (8.0 × 10^9^). In the literature [13], a TPU synthesized with similar components to those used in our study (MDI as isocyanate, macrodiol as polyol and diol as chain extender) and with a similar HS% (48 wt.% HS) was foamed in one-step, and it presented a heterogeneous cellular structure with the smallest cell size of ca. 0.5 µm, its relative density was 0.91 and the cell density was 4 × 10^9^ cells/cm^3^.

## 4. Conclusions

A series of TPU based on MDI, PEG-PPG-PEG and 1,5-PDO components with low contents of HS (40, 50 and 60 wt.%) are synthesized using the pre-polymer method. These TPUs have been characterized in detail by measuring their densities, Shore hardness, GPC, DSC, WAXD, DMTA, and rheology.

The foaming window for these materials in the one-step gas dissolution process has been evaluated at different saturation and foaming conditions. Initially, the effect of the foaming temperatures from 140 to 180 °C maintaining a saturation pressure of 20 MPa and a foaming time of 1 h has been evaluated for all the TPU grades. The results show that the optimum HS content is 50 wt.% because higher or lower HS ratios do not lead to TPU foams. In addition, the foaming conditions to obtain TPU foams with 50 wt.% HS have been optimized by varying the saturation pressure from 10 to 25 MPa, while maintaining the optimum foaming temperature (170 °C), and the foaming time (1 h). The best foaming conditions are 170 °C, 25 MPa and 1 h, since a TPU foam with the lowest relative density (0.74), the smallest average cell size (4 microns), and an improved cell nucleation density of 8.0 × 10^9^ cells/cm^3^ is obtained.

## Figures and Tables

**Table 1:** The amount of each component used for the first and second step of TPU synthesis.

	First Step		Second Step		
TPU Samples	MDI Isocyanate	PEG-PPG-PEG Polyol	Prepolymer	MDI Isocyanate	1,5-PDO Chain Extender
40% HS	43.8 g, 0.175 mol	85.8 g, 0.043 mol	90.6 g	0 g, 0 mol	9.4 g, 0.090 mol
50% HS	64.2 g, 0.257 mol	85.8 g, 0.043 mol	87.4 g	0 g, 0 mol	12.6 g, 0.121 mol
60% HS	64.2 g, 0.257 mol	85.8 g, 0.043 mol	69.9 g	13.4 g, 0.054 mol	16.7 g, 0.160 mol

**Table 2:** Properties of TPU samples: the density of the solid TPU (*ρ*s), Shore hardness, number-averaged molecular weights (*M*_n_), weigh-averaged molecular weights (*M*_w_) and polydispersity index (PDI).

TPU Samples	*ρ_s_* (Kg/m^3^)	Hardness (Shore A)	Hardness (Shore D)	*M*_n_ (g/mol)	*M*_w_ (g/mol)	PDI
40%HS	1.068 ± 0.002	78.8 ± 0.4	22.6 ± 0.5	18583 ± 1046	36556 ± 1576	1.97 ± 0.06
50%HS	1.125 ± 0.003	88.6 ± 0.5	37.6 ± 0.9	26403 ± 1469	51468 ± 1274	1.95 ± 0.06
60%HS	1.101 ± 0.003	-	45.6 ± 0.5	8552 ± 1602	18325 ± 535	2.18 ± 0.32

**Table 3:** DSC data of solid TPUs with different HS contents.

TPU Samples	*T*_g_ SS (°C)	*T*_m_ 1 (°C)	*T*_m_ 2 (°C)	*T*_m_ 3 (°C)	HS Crystallinity (%)
40%HS	−42	133	168	-	7.0
50%HS	−42	135	-	177	8.9
60%HS	−39	143	172	178	9.4

**Table 4:** DMTA data of solid TPUs with different HS contents.

TPU Samples	*T*_g_ SS (°C)	Storage Modulus at −100 °C (MPa)	Storage Modulus at 25 °C (MPa)
40%HS	−25.3 ± 0.9	3333 ± 667	43 ± 9
50%HS	−16.7 ± 0.3	3562 ± 423	50 ± 13
60%HS	−22.4 ± 0.1	4203 ± 214	197 ± 35

## Data Availability

Not applicable.
